# Strain-Dependent Cross-Neutralization of Closely Related Asian-Lineage Zika Viruses by Dengue-Immune Sera

**DOI:** 10.3390/biom16101386

**Published:** 2026-09-23

**Authors:** Jia-Yi Tan, Nurhafiza Zainal, Sazaly AbuBakar, Kim-Kee Tan

**Affiliations:** 1 Tropical Infectious Diseases Research and Education Centre (TIDREC), Higher Institution Center of Excellence (HICoE), Universiti Malaya, Kuala Lumpur 50603, Malaysia; tanjiayi@um.edu.my (J.-Y.T.); sazaly@um.edu.my (S.A.); 2 Department of Medical Microbiology, Faculty of Medicine, Universiti Malaya, Kuala Lumpur 50603, Malaysia; nurhafizazainal@um.edu.my; 3 Department of Biomedical Science, Faculty of Medicine, Universiti Malaya, Kuala Lumpur 50603, Malaysia

**Keywords:** antibodies, arbovirus, dengue virus, flavivirus, immunity, zika virus, infectious diseases

## Abstract

**Background**: Zika virus (ZIKV) and dengue virus (DENV) co-circulate in tropical regions and elicit cross-reactive immunity. However, differential neutralization of closely related ZIKV strains remains unclear. This study characterizes the neutralization profiles of dengue-immune sera against DENV and two Asian-lineage ZIKV strains, P6-740 and PRVABC59. **Methods**: Sera from suspected dengue infections and from healthy donors were screened for anti-DENV IgM and IgG antibodies and anti-ZIKV IgM and IgG antibodies by ELISA and grouped. Neutralization against DENV serotypes and two ZIKV strains was quantified using the foci reduction neutralization test (FRNT). Complete genome sequences of ZIKV P6-740 and PRVABC59 were compared to identify amino acid differences. **Results**: Sera containing anti-DENV IgG showed higher neutralizing activity against both DENV and ZIKV than IgG-negative sera. Neutralization was also detected in some ELISA-seronegative samples. Across all immunological groups, neutralization titers were higher against ZIKV P6-740 than ZIKV PRVABC59. Among healthy donors with evidence of previous dengue exposure, 58% exhibited a ≥4-fold difference between the two ZIKV strains, of which 80% favored ZIKV P6-740. Among seronegative healthy blood donors, 45% exhibited a 4-fold difference, of which 89% favored P6-740. Four amino acid substitutions were identified in the envelope (E) protein. **Conclusions**: Dengue-induced immunity differentially neutralizes closely related ZIKV strains, with generally stronger activity against P6-740, suggesting that viral genetic variation may influence cross-neutralization outcomes.

## 1. Introduction

Zika virus (ZIKV) and dengue virus (DENV) are mosquito-borne orthoflaviviruses transmitted primarily by *Aedes* mosquitoes. Both viruses share substantial genetic and structural similarities but differ markedly in their epidemiology and clinical impact. Dengue is one of the most prevalent mosquito-borne infections worldwide, causing an estimated 390 million infections annually [1]. In contrast, ZIKV was historically associated with only sporadic human cases [2] before the epidemic in the Americas, which was linked to Guillain–Barré syndrome (GBS) among adults and microcephaly among newborns [3]. The antigenic relationships among orthoflaviviruses have been investigated for decades. Early studies using hemagglutination inhibition and neutralization assays established extensive serological cross-reactivity among flaviviruses and contributed to their antigenic classification [4,5,6,7,8]. The antigenic cross-reactivity between DENV and ZIKV has prompted considerable interest in understanding whether immune responses induced by one virus may influence infection and immunity to the other. In most of Southeast Asia (SEA), DENV is hyperendemic, with all four DENV serotypes co-circulating [4]. In contrast, although ZIKV has been detected in the region for decades, large-scale outbreaks were not reported until 2016, when the first significant ZIKV-associated outbreak in SEA was reported in Singapore [5]. In Malaysia, ZIKV was first isolated from mosquitoes in Bentong, Pahang, in 1966 [6]. Subsequent surveillance [7,8] and serological studies suggest low-level circulation of the virus, despite the absence of major outbreaks [9,10,11]. The discrepancy between the widespread circulation of DENV and the limited spread of ZIKV in SEA and Malaysia, despite the widespread presence of the Aedes mosquitoes, raises the possibility of interference from pre-existing population immunity against DENV affecting the transmission of ZIKV.

Growing evidence suggests that antibodies induced by DENV infection can cross-react with and neutralize ZIKV, potentially providing some degree of cross-protection against subsequent infection [12]. This cross-neutralization is largely mediated through the recognition of conserved epitopes within the viral envelope (E) protein, which plays a crucial role in viral entry and serves as the primary target for neutralizing antibodies [13,14]. More recent studies have identified and mapped cross-reactive and cross-neutralizing epitopes on the flavivirus E protein [15,16,17], including conserved fusion loop regions within domain II and quaternary epitopes formed across adjacent E proteins [16,17,18]. Most studies investigating DENV-ZIKV cross-neutralization focused on single ZIKV strain or comparison between major viral genotypes [19,20]. However, the extent to which dengue-derived cross-neutralization differs among closely related ZIKV strains within the same lineage remains underexplored. Given that amino acid variation within structural proteins, particularly the E protein, can influence antibody recognition and neutralization sensitivity [21], strain-specific differences may represent an important but underappreciated determinant of flavivirus cross-reactivity. To address this gap, we investigated the neutralization profiles of dengue-immune sera against DENV and two Asian-lineage ZIKV strains: P6-740, an early Malaysian isolate representing one of the earliest recognized Asian-lineage viruses [6], and PRVABC59, a contemporary epidemic strain isolated during the 2015 Zika outbreak in Puerto Rico [22]. In addition, we compared the genome sequences of these strains to identify amino acid differences within the structural proteins that may be associated with differential neutralization. Understanding the extent and determinants of strain-dependent cross-neutralization may provide new insights into flavivirus immunity and improve the interpretation of serological and neutralization studies in dengue-endemic regions.

## 2. Materials and Methods

### 2.1. Cell Lines and Viruses

Two Asian-lineage ZIKV strains were used in this study. The ZIKV P6-740 strain, isolated in Malaysia in 1966, was obtained from Dr. Robert Tesh (World Reference Center for Emerging Viruses and Arboviruses, The University of Texas Medical Branch, Galveston, TX, USA) and represents one of the earliest identified Asian-lineage strains. PRVABC59 was isolated during the 2015 Zika outbreak in Puerto Rico and was purchased from the American Type Culture Collection (ATCC, Manassas, VA, USA; Cat. No. VR-1843; Zika virus [ATCC VR-1843]). Three DENV serotypes (DENV-1, DENV-2, and DENV-3) were represented by clinical isolates obtained from patients in Malaysia.

Vero cells (ECACC 88020401), provided by Prof. Peter Liljeström (Karolinska Institutet, Stockholm, Sweden), were used for ZIKV propagation, the foci forming assay (FFA), and the foci reduction neutralization test (FRNT), while C6/36 cells (ATCC CRL1660, American Type Culture Collection, Manassas, VA, USA) were used for DENV propagation. Vero cells were cultured in Dulbecco’s Modified Eagle’s medium (DMEM) with 10% fetal bovine serum (FBS; Bovogen, Keilor East, Australia) and 2 mM L-Glutamine at 37 °C with 5% carbon dioxide (CO_2_), whereas C6/36 cells were maintained in Eagle’s minimum essential medium (EMEM) with 10% FBS at 28 °C with 5% CO_2_. After infection, cells were incubated for 3 days (ZIKV) or 7 days (DENV). The culture supernatants were then harvested, filtered, and stored at −80 °C until needed. Virus titer determination was performed using an FFA, with the resulting titers expressed as foci-forming units per milliliter (FFU/mL).

### 2.2. Human Sera and Antibody Detection Methods

Sera samples were obtained from the repository at the Tropical Infectious Diseases Research and Education Centre (TIDREC). Dengue-specific immunoglobulin M (IgM) and immunoglobulin G (IgG) antibodies in the serum samples were detected using capture Enzyme-Linked Immunosorbent Assay (ELISA) kits (Standard Diagnostics, Yongin, Republic of Korea), while ZIKV antibodies were identified using Anti-ZIKV IgM and IgG ELISA kits (Euroimmun, Lüebeck, Germany). All ELISA procedures were performed according to the manufacturers’ protocols.

### 2.3. Foci Reduction Neutralization Test (FRNT)

The FRNT was used to assess the neutralization capacity of dengue-immune sera against ZIKV and DENV strains using Vero cells as described in Section 2.2. Virus stocks were quantified using a foci forming assay (FFA) as described [23] and standardized to 120 FFU per inoculum for all strains.

Heat-inactivated sera (56 °C, 30 min) from healthy donor sera were initially diluted 1:10 and sera from suspected dengue patients 1:20, followed by two-fold serial dilutions. Each serum dilution (250 µL) was mixed with an equal volume of virus (120 FFU) and incubated at 37 °C for 1 h to allow antibody–virus interaction. The virus–serum mixture was then added to a Vero cell monolayer that seeded 24 h prior to infection (80–90% confluency), followed by 1 h incubation at room temperature with gentle rocking to allow virus adsorption. After two washes of dPBS, infected cells were overlaid with DMEM containing 2% FBS and 1.5% CMC and incubated at 37 °C with 5% CO_2_ for foci development: incubation was 44 h for ZIKV PRVABC59, 60 h for ZIKV P6-740, and 96 h for DENV based on previous optimization. Infected cells were fixed, permeabilized, and immunostained as previously described [23].

Neutralizing antibody titers (FRNT_50_) were determined as the reciprocal of serum dilution causing a 50% reduction in foci numbers. An FRNT_50_ of 1:40 was used as the assay cut-off, and samples with FRNT_50_ < 40 were considered non-reactive, while FRNT_50_ ≥ 40 reactive. Each serum dilution was tested in duplicate wells within a single FRNT run, and the mean foci count from duplicate wells was used to calculate FRNT_50_.

### 2.4. RNA Extraction, Viral Genome Fragments Amplification, and Sequencing

Viral ribonucleic acid (RNA) was extracted from the supernatant of infected cell culture using the QIAamp Viral RNA Mini Kit (Qiagen, Hilden, Germany) according to the manufacturer’s protocol. ZIKV RNA was reverse-transcribed into complementary deoxyribonucleic acid (cDNA) using a specific 3′ untranslated region (UTR) primer. The complete ZIKV genome was amplified into seven overlapping fragments using DreamTaq DNA Polymerase (Thermo Fisher Scientific, Waltham, MA, USA), following established protocols [24,25] with minor modifications. Amplified polymerase chain reaction (PCR) products were purified using the QIAquick Gel Extraction Kit (Qiagen, Germany) as stated in the manufacturer’s protocol and sequenced by Sanger sequencing. The sequencing reads generated were assembled using Sequencher^®^ v5.0 (Gene Codes Corporation, Ann Arbor, MI, USA) and assembled using the reference assembly method.

### 2.5. Statistical Analysis

Prism v9.0.0 (GraphPad, Boston, MA, USA) was used for statistical analysis. Geometric mean titers (GMTs) were calculated [26]. For GMT calculations, a FRNT_50_ value of 10 was assigned to samples with titers below the lowest dilution tested. The lowest final serum dilution was 1:20 for healthy blood donor serum samples and 1:40 for suspected dengue serum samples because of limited serum availability. Differences in neutralization titers among the different viruses within each immunological serum group were analyzed using the Friedman test, followed by Dunn’s multiple comparisons test. A two-sided *p*-value of ≤0.05 was considered statistically significant.

## 3. Results

### 3.1. Sample Characterization

A total of 215 serum samples from individuals with suspected dengue virus infection were screened for the presence of anti-DENV and anti-ZIKV antibodies. Among these, 49 samples (22.8%) were positive for anti-DENV antibodies but negative for anti-ZIKV antibodies. A total of 11 were anti-DENV IgM-positive only, 31 were anti-DENV IgG-positive only, and 7 were positive for both anti-DENV IgM and IgG, suggesting a recent infection. Based on these serological profiles and sample availability, sera representing recent dengue infection (anti-DENV IgM+/IgG+) and those with anti-DENV IgM+/IgG- were used for virus neutralization studies.

For the healthy blood donors, 215 archived sera were used for screening. Based on available serum volume and initial DENV IgM and IgG serology results, 63 serum samples were selected for use in the ELISA. Results from the ELISA showed that 43 (68.3%) were positive for anti-DENV IgG only, while 20 (31.7%) were negative for both anti-DENV IgM and IgG. All selected donor samples were then used for further analysis, and those samples were negative for anti-ZIKV IgM and IgG antibodies.

Collectively, the final study cohort comprised four groups: seronegative healthy donors (dDENV_IgM-/IgG-), healthy donors with evidence of past dengue exposure (dDENV_IgM-/IgG+), suspected dengue patients with anti-DENV IgM only (dDENV_IgM+/IgG-), and suspected dengue patients positive for both anti-DENV IgM and IgG (sDENV_IgM+/IgG+). The final number of samples in each group is summarized in Table 1.

### 3.2. Cross-Neutralization of DENV and ZIKV by Sera from Different Immune Groups

Neutralization capacity against DENV and ZIKV was assessed using FRNT_50_ and expressed as GMT across the four immune groups (Figure 1). Due to limited serum availability and the low prevalence of DENV-4 in Klang Valley, Malaysia, healthy blood donor samples were tested against DENV-1, DENV-2, and DENV-3 only. Samples of suspected dengue patients were tested against DENV-1 and DENV-3 only.

Among healthy blood donors without detectable anti-DENV IgM or IgG (dDENV_IgM-/IgG-; Figure 1A), neutralization titers differed significantly among the five viruses (Friedman test, *p* < 0.0001). DENV-1 showed significantly higher titers than DENV-2, DENV-3, and ZIKV PRVABC59 (Dunn’s adjusted *p* = 0.0001, 0.0270, and <0.0001, respectively). In the dDENV_IgM-/IgG+ group (Figure 1B), neutralization titers also differed significantly among the five viruses (Friedman test, *p* < 0.0001). Neutralization titers of ZIKV P6-740 were significantly higher than those against ZIKV PRVABC59 (adjusted *p* = 0.0336). Among suspected dengue patients, neutralization titers differed significantly among the four viruses tested in both sDENV_IgM+/IgG- (Figure 1C; Friedman test, *p* = 0.0003) and sDENV_IgM+/IgG+ group (Figure 1D; Friedman test, *p* = 0.0395). DENV-1 showed significantly higher titers than ZIKV PRVABC59 in the sDENV_IgM+/IgG- group (Dunn’s adjusted *p* = 0.0016), whereas none of the pairwise comparisons remained significant after correction in the sDENV_IgM+/IgG+ group.

Overall, neutralization profiles varied among the viruses tested within each immune group. Neutralization titers against ZIKV P6-740 were higher than those against PRVABC59 across all four immune groups, although the difference was statistically significant only in the dDENV_IgM-/IgG+ group.

### 3.3. Strain-Dependent Neutralization of ZIKV P6-740 and PRVABC59

To further investigate whether dengue-associated immunity differentially neutralized ZIKV strains, paired FRNT_50_ titers against ZIKV P6-740 and ZIKV PRVABC59 were compared using healthy donors’ sera (Figure 2). A 4-fold difference threshold was applied to identify biologically meaningful differences in neutralization between the two virus strains.

Among healthy blood donors with evidence of prior dengue exposure (dDENV_IgM-/IgG+), comparative analysis revealed distinct differences in neutralization titers, of which 58% (25/43) of sera exhibited a ≥4-fold difference in neutralization between ZIKV P6-740 and ZIKV PRVABC59 (Figure 2A). The majority (80%, or 20/25) of these samples preferentially neutralized ZIKV P6-740 over ZIKV PRVABC59. Similarly, among the seronegative healthy donors’ group (dDENV_IgM-/IgG- group), 45% (9/20) of sera showed ≥4-fold differences, of which 89% (8/9) preferentially neutralized ZIKV P6-740 and 11% (1/9) preferentially neutralized ZIKV PRVABC59 (Figure 2B). Overall, across both healthy donor groups, only 6 of 34 sera (18%) with ≥4-fold differences preferentially neutralized ZIKV PRVABC59, whereas 28 of 34 sera (82%) preferentially neutralized ZIKV P6-740. The predominance of sera preferentially neutralizing P6-740 suggested that the observed differences were likely directional rather than random. The complete individual serum neutralization profiles for dDENV_IgM-/IgG- and dDENV_IgM-/IgG+ groups are provided in Supplementary Materials 1 and 2, respectively.

### 3.4. Genetic Differences in Structural Proteins (prM and E) Between ZIKV P6-740 and ZIKV PRVABC59

To investigate potential genetic differences associated with the observed strain-dependent neutralization patterns, the complete genome sequences of ZIKV P6-740 and PRVABC59 were compared. Overall, the two strains differed by 1.17% at amino acid level. Given the role of structural proteins in antibody recognition, amino acid substitutions in the prM and E proteins were further examined (Table 2). Five amino acid substitutions were identified in the prM protein at position 1 (V/A), 17 (S/N), 21 (K/E), 31 (V/M) and 109 (S/P), corresponding to the highest amino acid substitution rate among all viral proteins (2.98%). In addition, four amino acid substitutions were identified in the E protein at positions E-156 (I/T), E-393 (D/E), E-473 (V/M), and E-487 (T/M). The identified E protein substitutions were located within domains I and III, and the C-terminal transmembrane anchor regions.

## 4. Discussion

In this study, we investigated neutralization profiles of dengue-immune sera against DENV serotypes and two Asian-lineage ZIKV strains including ZIKV PRVABC59, which was associated with the 2016 pandemic [22], and ZIKV P6-740, a historical Malaysian isolate obtained in 1966 [6]. Results from the study revealed that sera with anti-DENV IgG displayed multitypic neutralization against DENV-1, DENV-2, and DENV-3, and a substantial proportion of samples (50.0–74.4%) demonstrated cross-neutralizing activity against ZIKV strains. This is consistent with previous reports that sera from individuals with evidence of prior dengue exposure can bind and neutralize ZIKV [27]. We observed that substantial differences occurred in cross-neutralization efficiencies of dengue-immune sera against the two Asian-lineage ZIKV strains. Across the immune groups, neutralization titers against ZIKV P6-740 were generally higher than that against ZIKV PRVABC59, with a substantial proportion of sera demonstrating at least 4-fold differences in neutralization titers between the two strains. However, detectable cross-neutralization does not necessarily indicate highly potent neutralizing activity, as ZIKV neutralization titers were generally lower compared with those observed against DENV serotypes. Our findings suggest that the pre-existing dengue immunity may contribute to ZIKV cross-neutralization in this Malaysian cohort, and the cross-neutralization may vary between closely related ZIKV strains.

Pre-existing dengue immunity in endemic regions is complex and can be shaped by multiple factors, including infecting serotype [28,29,30], number of exposures [31], and the sequence and timing of infections [32,33]. The relatively high neutralization titers against DENV-1 observed across the different serological groups may reflect the epidemiological history of dengue in the study population. DENV-1 has prolonged circulation in the Klang Valley [4], and previous population exposure may have contributed to this pattern, although individual histories of infecting DENV serotypes were unavailable. The contribution of individual antibody isotypes may also vary over the course of infection, with IgM reported to contribute substantially to flavivirus neutralization during the early antibody response [34]. In our study, neutralization titers in the IgM-only group were broadly similar to those observed in seronegative group across the viruses tested. As IgM-mediated neutralizing responses have been reported to vary over the course of infection, the timing of sample collection could have influenced the observed neutralization titers. Due to the unavailability of days after symptom onset for the suspected dengue samples, we were unable to determine whether the timing of sample collection contributed to this observation. Together, these factors may contribute to substantial variation in the breadth and potency of specific and cross-reactive immune responses among individuals. Cross-reactive immunity between DENV and ZIKV has been widely documented due to high sequence similarity shared among flaviviruses [27]. Previous studies have shown that antibodies induced by DENV infection can bind and neutralize ZIKV [19,20,35]. However, the interaction between DENV and ZIKV is complex, as the magnitude and durability of the cross-reactivity are highly variable across cohorts and immune histories [19,36]. Although some studies showed that DENV-induced ZIKV cross-neutralizing activity is often limited and not uniformly durable, especially several months after infection [19,35], others have shown that cross-reactive flavivirus antibodies may persist long-term [36]. In our study, a substantial proportion (approximately 30–60% depending on immune groups) of individuals demonstrated measurable cross-neutralization against one or both ZIKV strains. This observation supports the possibility that repeated DENV exposure may contribute to higher ZIKV neutralizing titers [37].

Earlier studies of cross-neutralization of ZIKV by dengue-immune sera have focused on comparisons between African and Asian/American genotypes [38,39,40,41], and relatively few have examined neutralization differences among closely related strains within the same lineage [19,20]. In the present study, we showed that despite belonging to the same Asian lineage, ZIKV P6-740 and ZIKV PRVABC59 exhibited markedly different neutralization profiles when tested against dengue-immune sera. Around 45–60% of serum samples exhibited at least a 4-fold difference in neutralization between the two strains, with the majority showing stronger neutralization against ZIKV P6-740. A minority of sera preferentially neutralized ZIKV PRVABC59, and some sera demonstrated discordant patterns, efficiently neutralizing one strain while exhibiting minimal neutralizing activity against the other. These findings suggest that strain-specific characteristics may influence the magnitude of dengue-derived cross-neutralization against different ZIKV strains. The preferential neutralization of ZIKV P6-740 may reflect antigenic differences between the two strains, but it may also be influenced by the epidemiological history of ZIKV circulation in Malaysia. ZIKV P6-740 represents a historical Malaysian isolate [6] obtained decades before the 2016 epidemic, and related Asian-lineage viruses may have circulated undetected in Southeast Asia for many years. Since most ZIKV infections are asymptomatic [42], early-life exposure to ZIKV or antigenically related flaviviruses cannot be excluded. Therefore, repeated exposure to locally circulating ZIKV strains that are more closely related to P6-740 may have contributed to the stronger neutralization observed against this strain. However, because individual ZIKV infection histories were unavailable, the contribution of previous asymptomatic ZIKV exposure remains speculative.

Comparable findings have been reported for ZIKV, where serum raised against the African-lineage ZIKV MR-766 neutralized the Asian-lineage ZIKV H/PF/2013 strain, but with lower titers, highlighting variability in neutralization across ZIKV strains [38]. Notably, MR-766 lacks the N-linked glycosylation site at position N154 of the E protein, which is conserved in most Asian-lineage strains, representing one of the structural differences that may contribute to differences in antigenic recognition between these viruses [43]. Within DENV, several studies have also demonstrated genotype-dependent differences in neutralization even among the viruses belonging to the same serotype or genotype [28,29]. Differences in local DENV serotype dominance, patterns of repeated infection, and the neutralization assay used (including the virus strain and readout method used) [44] are likely to contribute to these divergent observations across studies. Our study further suggests that interpretations regarding flavivirus cross-protection may depend in part on the ZIKV strains included in the study. These results highlight viral strain selection as an important but often underappreciated variable when evaluating virus cross-reactivity and cross-protection.

To explore potential impact of genome variation associated with the strain-dependent neutralization difference observed in this study, we compared the genome sequences of ZIKV P6-740 and ZIKV PRVABC59, particularly focusing on the E protein, likely the key factor associated with differential neutralization profiles between ZIKV strains [45,46,47]. These two strains differ in four positions in the E protein (E-156, E-393, E-473, and E-487). Three of the four E-protein substitutions identified between P6-740 and PRVABC59 (D393E, V473M, T487M) have previously been reported during the evolution of Asian-lineage ZIKV. E-D393E and E-T487M were detected in Asian-lineage viruses circulating in Asia and the Pacific between 2006 and 2013, whereas E-V473M was first detected in 2013 and subsequently became prevalent among the epidemic-associated strains [48].

Notably, E-156 lies within the N-linked glycosylation motif (positions 154–156), which is critical for virion assembly, infectivity, and vector competency [49,50]. Our sequence analysis revealed that the N-linked glycosylation motif at E-154–156 is maintained in ZIKV PRVABC59, with threonine (T) at position E-156 preserving the N-X-S/T motif, whereas ZIKV P6-740 contains isoleucine (I) at E-156, resulting in disruption of the N-linked glycosylation motif and loss of the glycosylation site. This motif is highly conserved among Asian-lineage ZIKV strains, which predominantly retain T at E-156. Mutations that disrupt this motif have been reported to affect neutralizing epitope accessibility and antibody recognition that potentially affect neutralization sensitivities [51,52]. E-393 is located within the FG-loop of domain III, a lateral ridge epitope region targeted by human ZIKV-neutralizing antibodies [45]. The D393E mutation has been shown to modulate the binding and neutralization potency of the ZIKV monoclonal antibody, ZIKA-116 [45]. Although the contribution of E-393 variation to DENV-derived cross-neutralization remains unknown, its location within a known neutralizing epitope makes it a candidate for further investigation. Additional substitutions were identified within the transmembrane regions of the E protein including V473M. Although this substitution has been associated with increased virulence, intrauterine transmission, and urban transmission [21], these phenotypes are not necessarily mediated through altered antibody neutralization. Given its location within the transmembrane region, the contribution of E-473 variation to antibody recognition and neutralization remains unclear. Together, these observations support the possibility that a relatively small number of naturally occurring amino acid substitutions may contribute to strain-dependent neutralization patterns observed between ZIKV P6-740 and ZIKV PRVABC59. Future studies incorporating additional ZIKV strains or targeted mutagenesis approaches will be required to determine the contribution of individual amino acid substitutions to the observed neutralization differences.

An additional observation in this study was the detection of measurable neutralization activity among a subset of sera lacking detectable anti-DENV and anti-ZIKV IgM or IgG antibodies by ELISA. Several explanations are tenable. First, individuals may have been exposed to other flaviviruses like JEV, which can elicit cross-reactive antibodies that bind or neutralize heterologous flaviviruses such as DENV and ZIKV [53]. All sampling sites in this study were located in Peninsular Malaysia, where JEV vaccination is not part of the routine immunization program. In Malaysia, routine JEV vaccination is primarily implemented in Sarawak, where JEV is endemic [54]. However, sporadic JEV cases have been reported in Peninsular Malaysia [54]. Therefore, previous natural JEV exposure as well as JEV vaccination in individual participants cannot be completely excluded, as individual vaccination and infection histories were unavailable. As a result, some individuals may appear seronegative by DENV- and ZIKV-specific ELISAs but may still be capable of cross-neutralizing DENV and ZIKV. Second, previous ZIKV or DENV infections cannot be ruled out, especially those with low residual antibody levels or immune profiles that are not fully captured by commercial ELISA assays but can still produce measurable neutralization activity. The limited sensitivity and specificity of flavivirus ELISAs may therefore result in under-recognition of prior DENV or ZIKV infections, especially for individuals infected many years ago [55,56,57]. Although the origin of these responses could not be determined in this study, our findings highlight the complexity of interpreting flavivirus immunity in endemic settings.

One limitation of the present study is that ZIKV and DENV stocks were propagated in different cell lines (Vero and C6/36 cells, respectively), which may influence measured neutralization sensitivity and should therefore be considered when comparing neutralization titers between DENV and ZIKV. However, both ZIKV strains were propagated in Vero cells and evaluated using the same Vero cell FRNT platform, minimizing this source of variation in primary comparison between ZIKV P6-740 and ZIKV PRVABC59. Beyond antibody-mediated neutralization, future studies should also investigate the contribution of cellular immunity, particularly DENV- and ZIKV-specific T-cell responses, to flavivirus cross-protection. Cross-reactive CD4+ and CD8+ T-cell responses have been reported between DENV and ZIKV and may contribute to immune protection when neutralizing antibody responses are incomplete or decline over time. Integrating antibody, B-cell, and T-cell immune profiling will provide a more comprehensive understanding of the mechanisms underlying flavivirus cross-reactivity and protection.

## 5. Conclusions

In conclusion, our findings revealed that pre-existing dengue immunity in a Malaysian cohort is associated with detectable cross-neutralizing antibody responses against both ZIKV strains, although the magnitude of neutralization varies among immune groups. Neutralization of ZIKV varies between the two strains, with generally higher titers against the historic Malaysian strain P6-740 than against the epidemic strain PRVABC59, despite both belonging to the Asian lineage. Comparative genomic analysis identified amino acid substitutions in E proteins that may contribute to these strain-dependent neutralization patterns. The unexpected detection of neutralization activity in some ELISA-seronegative samples highlights the complexity of defining true seronegative status in flavivirus-endemic settings. This indicates the limitations of relying solely on binding assays to infer past DENV or ZIKV infections. Collectively, these findings emphasize that cross-neutralization between DENV and ZIKV is not uniform across viral strains, and both antigenic variation and local immunity landscapes must be considered when interpreting serological data and designing vaccines and diagnostic techniques for endemic regions with co-circulating flaviviruses.

## Figures and Tables

**Figure 1:**
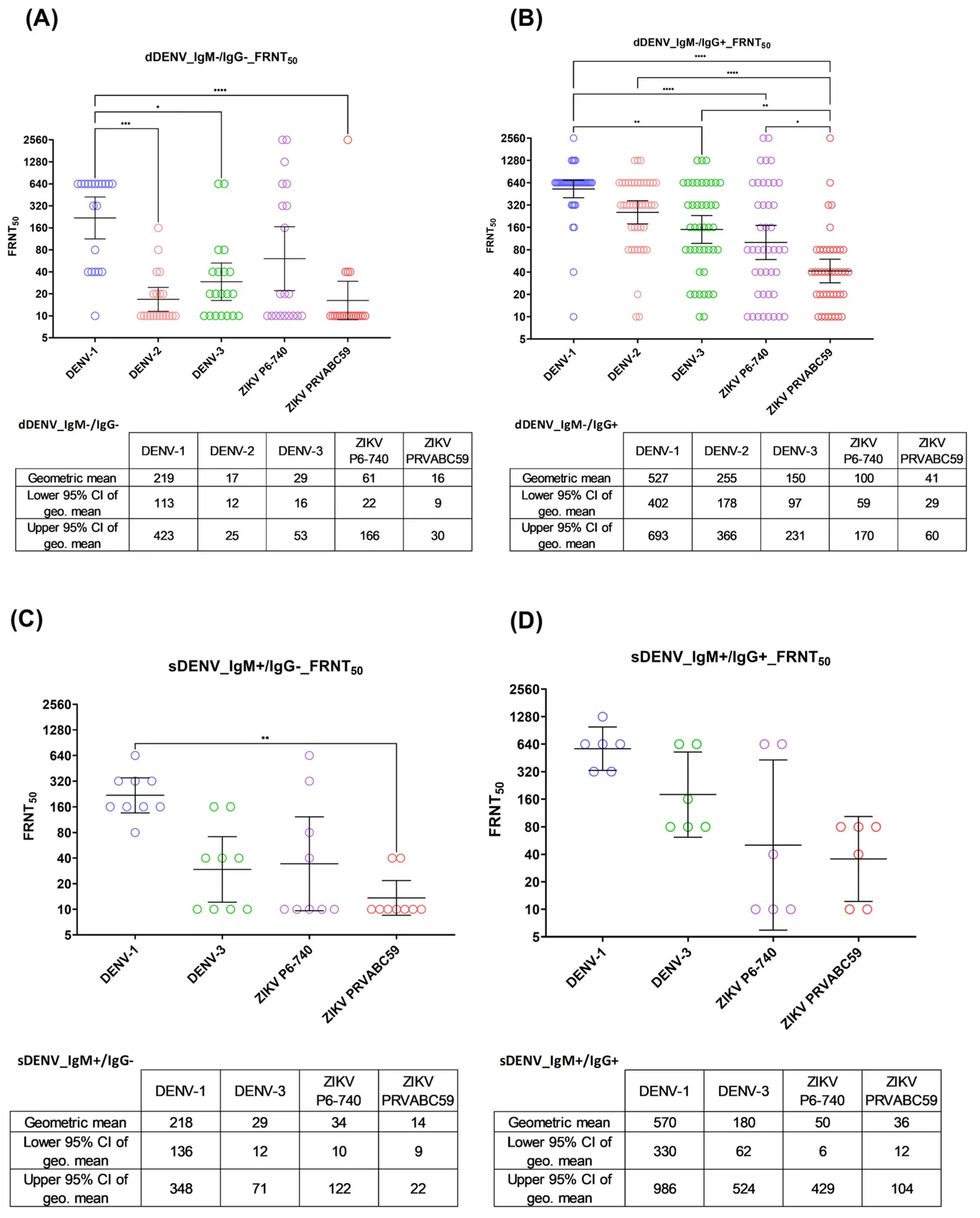
Neutralizing antibody titers (FRNT_50_) against DENV and ZIKV strains. Neutralization titers against DENV-1, DENV-2, DENV-3, ZIKV P6-740 and ZIKV PRVAC59 were determined by FRNT_50_. Results are shown for (**A**) dDENV_IgM-/IgG-, (**B**) dDENV_IgM-/IgG+, (**C**) sDENV_IgM+/IgG-, and (**D**) sDENV_IgM+/IgG+ immune groups. Each symbol represents an individual serum sample. Horizontal bars indicate geometric mean titers (GMTs), and error bars represent 95% confidence intervals. Statistical comparisons among viruses within each immune group were performed using the Friedman test with Dunns’s multiple comparison test. Significant differences are indicated as * *p* < 0.05, ** *p* < 0.01, *** *p* < 0.001, and **** *p* < 0.0001. Different colors represent the viruses tested: blue, DENV-1; green, DENV-3; purple, ZIKV P6-740; and red, ZIKV PRVABC59.

**Figure 2:**
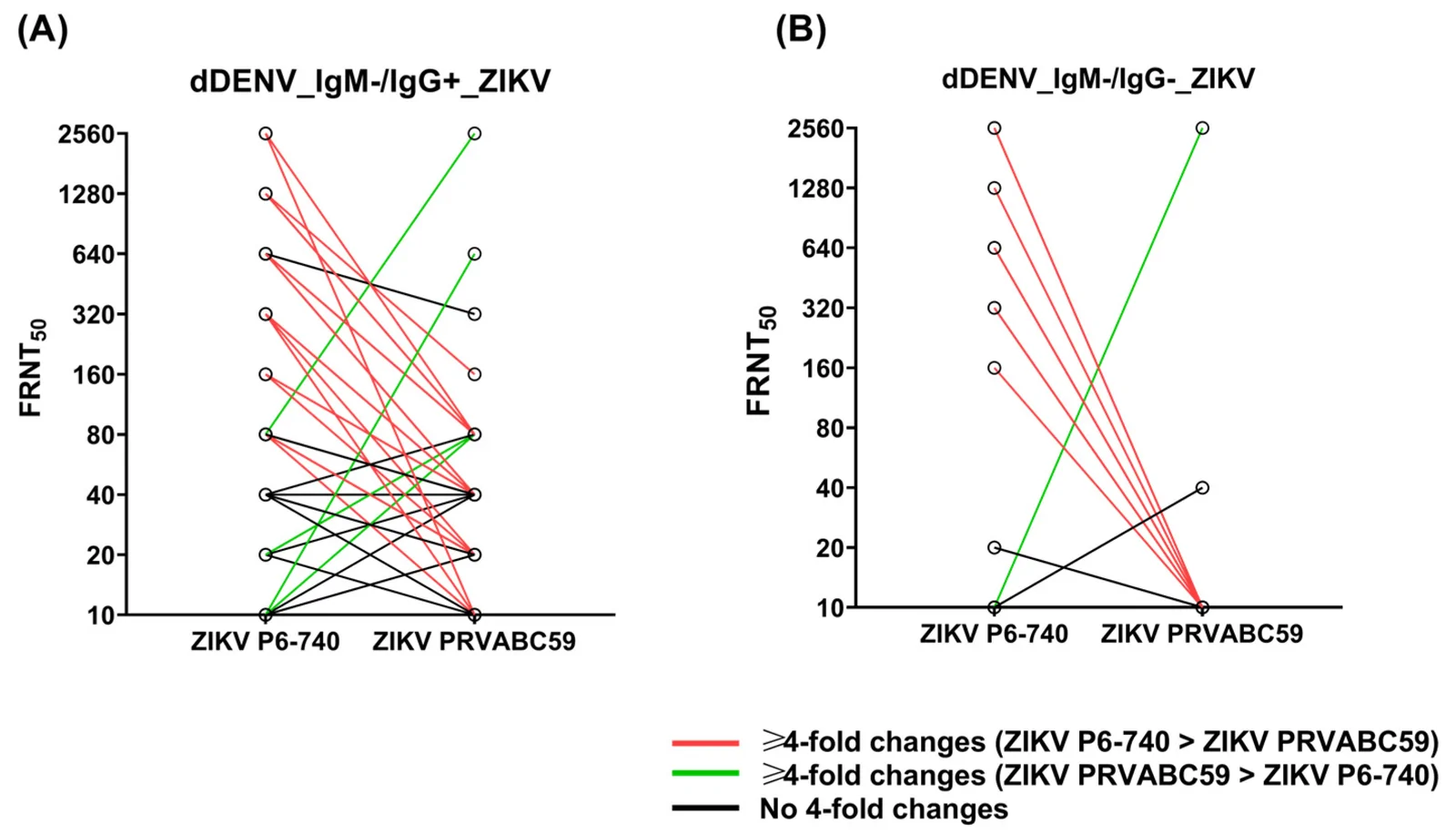
Differential neutralization of ZIKV P6-740 and ZIKV PRVABC59 by healthy donor sera. Paired FRNT_50_ titers against ZIKV P6-740 and ZIKV PRVABC59 were compared in (**A**) healthy blood donors with prior dengue exposure (dDENV_IgM-/IgG+ group) and (**B**) seronegative healthy donors (dDENV_IgM-/IgG-). Each point represents an individual serum sample; some data points overlap where sera have identical FRNT_50_ titers against the same ZIKV strain. Red lines indicate sera exhibiting ≥4-fold higher neutralization titers against ZIKV P6-740 than against ZIKV PRVABC59. Green lines indicate sera exhibiting ≥4-fold higher neutralization titers against ZIKV PRVABC59 than against ZIKV P6-740.

**Table 1:** Final study cohort stratification and sample numbers.

Study Group	Serological Profile	Final Numbers
Seronegative healthy donors	DENV IgM-/IgG-	20
Healthy donors with evidence of past dengue exposure	DENV IgM-/IgG+	43
Suspected dengue patients with anti-DENV IgM only	DENV IgM+/IgG-	11
Suspected dengue patients positive for both anti-DENV IgM and IgG	DENV IgM+/IgG+	7

**Table 2:** Amino acid substitutions identified in the structural proteins (prM and E) of ZIKV P6-740 and ZIKV PRVABC59.

Protein	Protein Position	ZIKV P6-740	ZIKV PRVABC59
**prM**	1	V	A
	17	S	N
	21	K	E
	31	V	M
	109	S	P
**E**	156	I	T
	393	D	E
	473	V	M
	487	T	M

## Data Availability

The ZIKV genome sequences generated in this study are available in the GISAID database under accession numbers EPI_ISL_20528963 and EPI_ISL_20528994.

## Supplementary Materials

The following supporting information can be downloaded at: https://www.mdpi.com/article/10.3390/biom16101386/s1, Supplementary Material S1: Individual Serum Neutralization Profiles of the dDENV_IgM-/IgG- Group; Supplementary Material S2: Individual Serum Neutralization Profiles of the dDENV_IgM-/IgG+ Group.

## Abbreviations

°C	Degrees Celsius
µL	Microliter
ATCC	American Type Culture Collection
CO_2_	Carbon dioxide
DAB	Diaminobenzidine
DENV	Dengue virus
DENV-1	Dengue virus type 1
DENV-2	Dengue virus type 2
DENV-3	Dengue virus type 3
DENV-4	Dengue virus type 4
DMEM	Dulbecco’s modified Eagle’s medium
DNA	Deoxyribonucleic acid
dPBS	Dulbecco’s phosphate-buffered saline
E	envelope
ELISA	Enzyme-linked immunosorbent assays
EMEM	Eagle’s minimum essential medium
FBS	Fetal bovine serum
FFA	Foci forming assay
FFU	Foci-forming units
FRNT	Foci reduction neutralization test
GBS	Guillain–Barre syndrome
GMT	Geometric mean titer
IgG	Immunoglobulin G
IgM	Immunoglobulin M
JEV	Japanese encephalitis virus
ml	Milliliter
NMRR	National Medical Research Register
PCR	Polymerase chain reaction
RNA	Ribonucleic acid
SEA	Southeast Asia
TIDREC	Tropical Infectious Diseases Research and Education Centre
USA	United States of America
UTR	Untranslated region
ZIKV	Zika virus
