# Supplementary material for: Strain-Dependent Cross-Neutralization of Closely Related Asian-Lineage Zika Viruses by Dengue-Immune Sera
Source: Biomolecules. 2026 Sep 23;16(10):1386. doi: 10.3390/biom16101386 (PMC13702172; doi:10.3390/biom16101386)

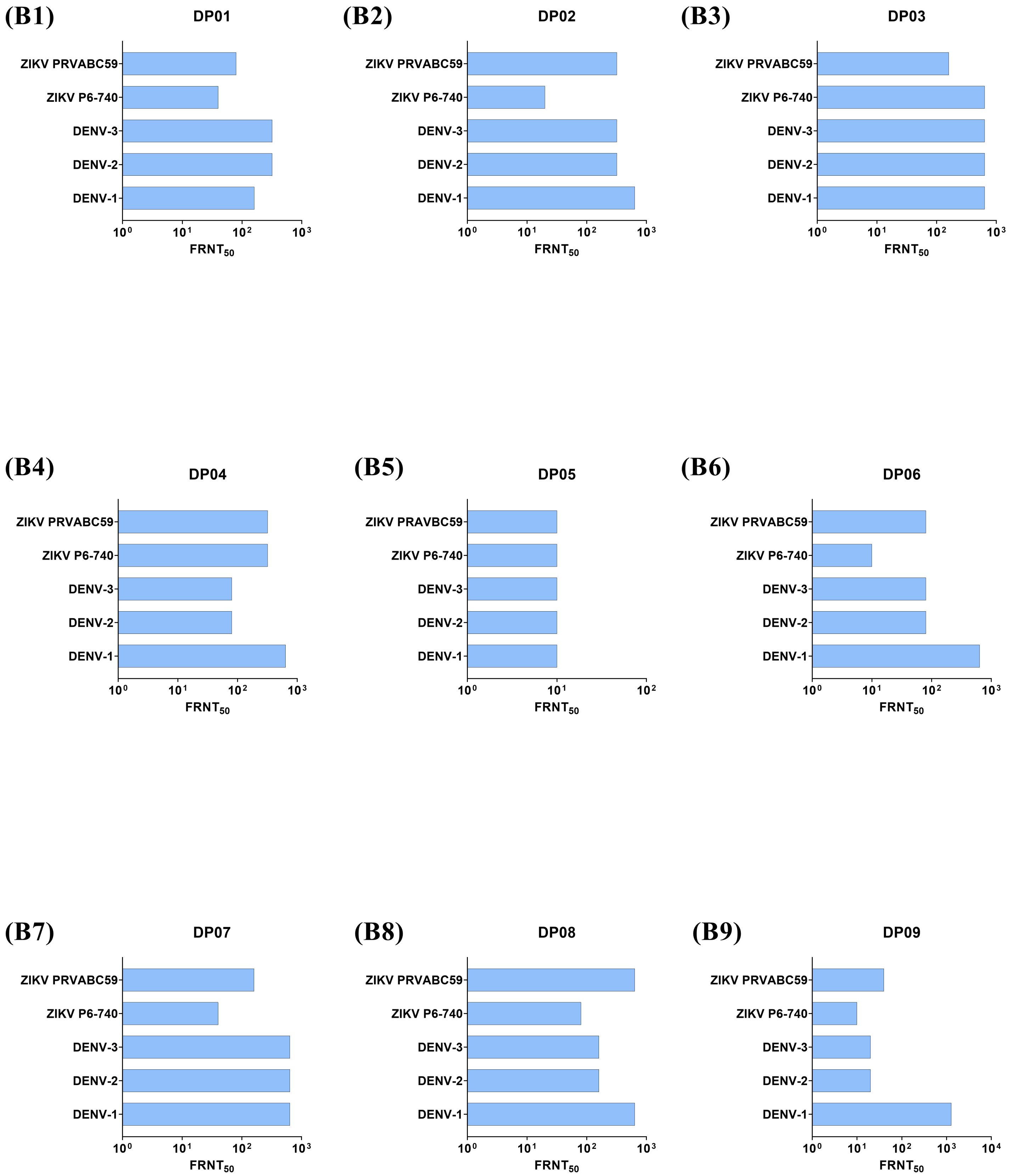

(B1)
DP01
ZIKV PRVABC59
ZIKV P6-740
DENV-3
DENV-2
DENV-1
FRNT50
(B2)
DP02
ZIKV PRVABC59
ZIKV P6-740
DENV-3
DENV-2
DENV-1
FRNT50
(B3)
DP03
ZIKV PRVABC59
ZIKV P6-740
DENV-3
DENV-2
DENV-1
FRNT50
(B4)
DP04
ZIKV PRVABC59
ZIKV P6-740
DENV-3
DENV-2
DENV-1
FRNT50
(B5)
DP05
ZIKV PRAVBC59
ZIKV P6-740
DENV-3
DENV-2
DENV-1
FRNT50
(B6)
DP06
ZIKV PRVABC59
ZIKV P6-740
DENV-3
DENV-2
DENV-1
FRNT50
(B7)
DP07
ZIKV PRVABC59
ZIKV P6-740
DENV-3
DENV-2
DENV-1
FRNT50
(B8)
DP08
ZIKV PRVABC59
ZIKV P6-740
DENV-3
DENV-2
DENV-1
FRNT50
(B9)
DP09
ZIKV PRVABC59
ZIKV P6-740
DENV-3
DENV-2
DENV-1
FRNT50

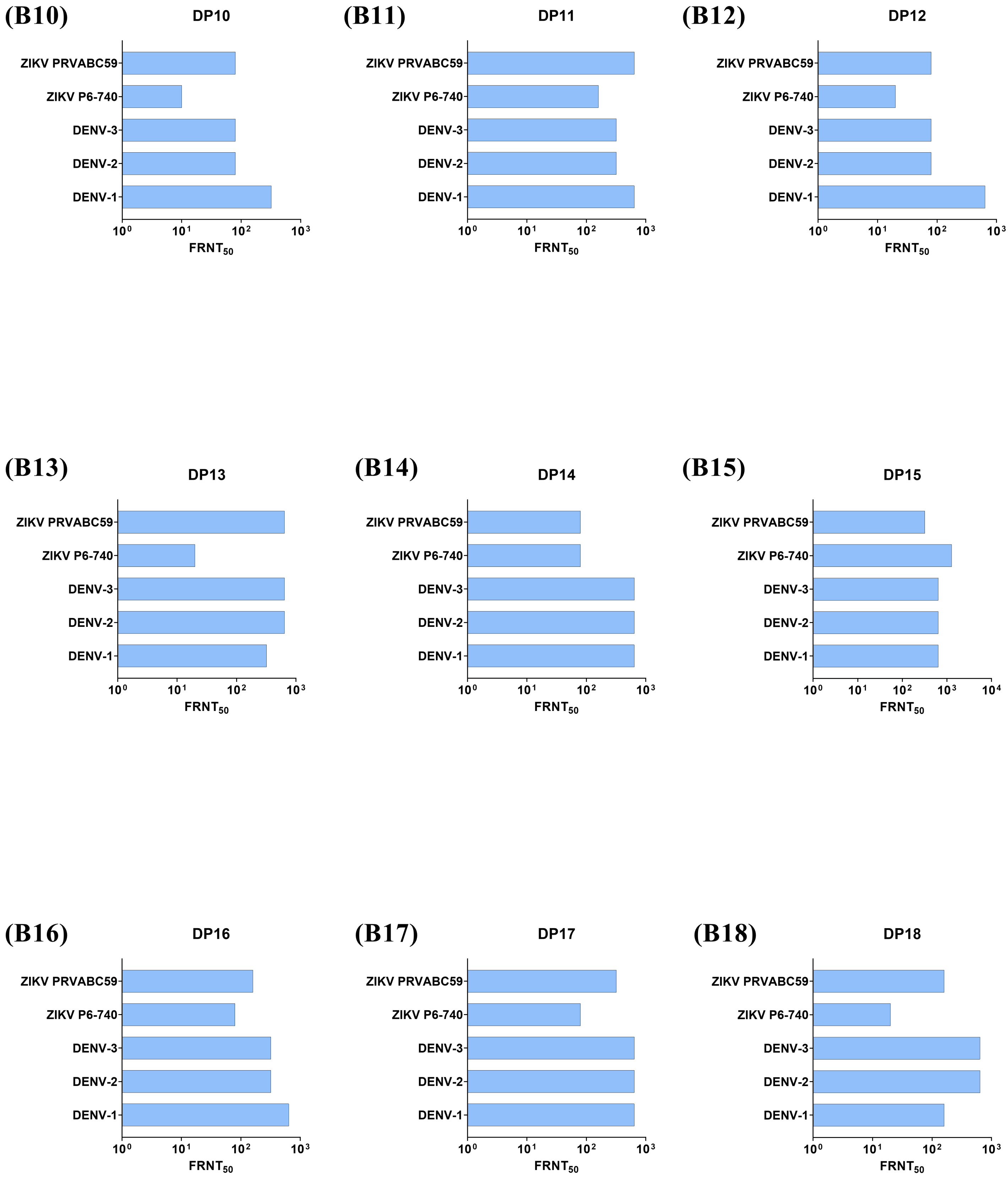
(B10)
DP10
ZIKV PRVABC59
ZIKV P6-740
DENV-3
DENV-2
DENV-1
10^0 10^1 10^2 10^3
$FRNT_{50}$
(B11)
DP11
ZIKV PRVABC59
ZIKV P6-740
DENV-3
DENV-2
DENV-1
10^0 10^1 10^2 10^3
$FRNT_{50}$
(B12)
DP12
ZIKV PRVABC59
ZIKV P6-740
DENV-3
DENV-2
DENV-1
10^0 10^1 10^2 10^3
$FRNT_{50}$
(B13)
DP13
ZIKV PRVABC59
ZIKV P6-740
DENV-3
DENV-2
DENV-1
10^0 10^1 10^2 10^3
$FRNT_{50}$
(B14)
DP14
ZIKV PRVABC59
ZIKV P6-740
DENV-3
DENV-2
DENV-1
10^0 10^1 10^2 10^3
$FRNT_{50}$
(B15)
DP15
ZIKV PRVABC59
ZIKV P6-740
DENV-3
DENV-2
DENV-1
10^0 10^1 10^2 10^3 10^4
$FRNT_{50}$
(B16)
DP16
ZIKV PRVABC59
ZIKV P6-740
DENV-3
DENV-2
DENV-1
10^0 10^1 10^2 10^3
$FRNT_{50}$
(B17)
DP17
ZIKV PRVABC59
ZIKV P6-740
DENV-3
DENV-2
DENV-1
10^0 10^1 10^2 10^3
$FRNT_{50}$
(B18)
DP18
ZIKV PRVABC59
ZIKV P6-740
DENV-3
DENV-2
DENV-1
10^0 10^1 10^2 10^3
$FRNT_{50}$

(B19)

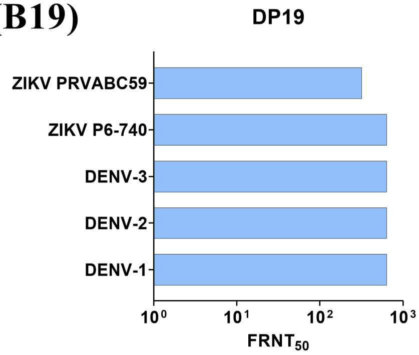

(B20)

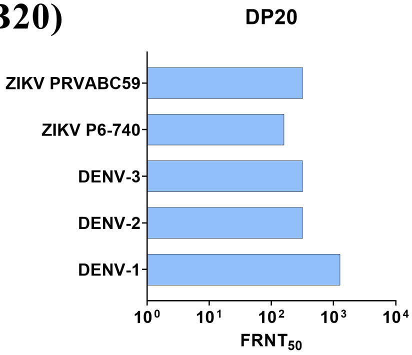

(B21)

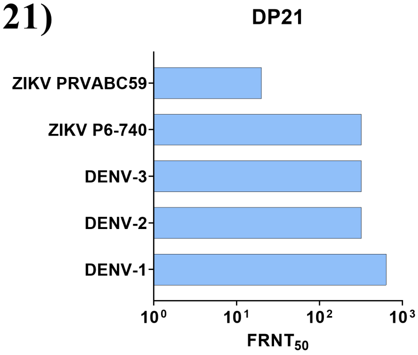

(B22)

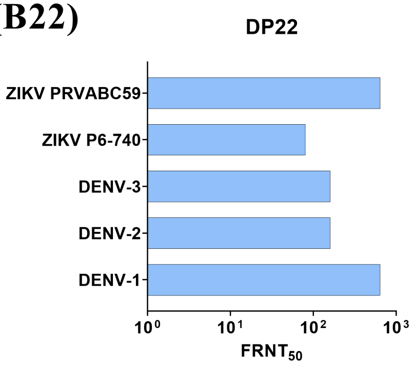

(B23)

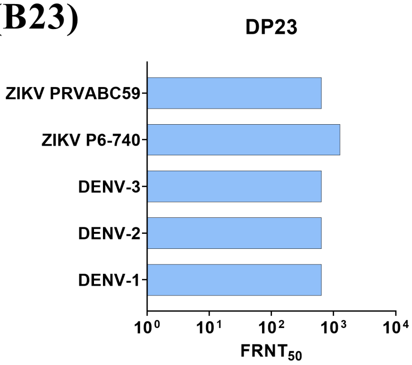

(B24)

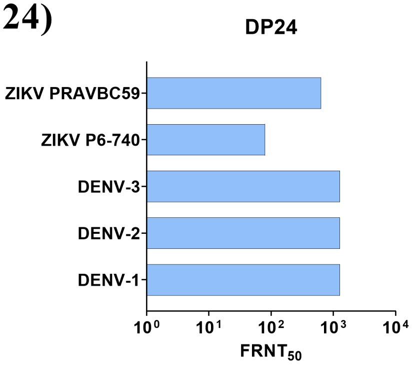

(B25)

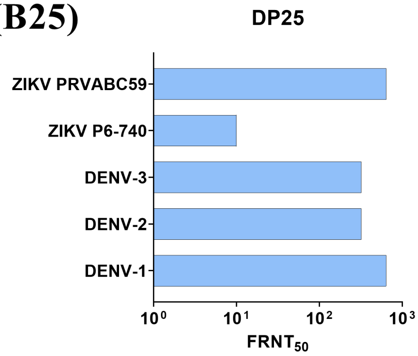

(B26)

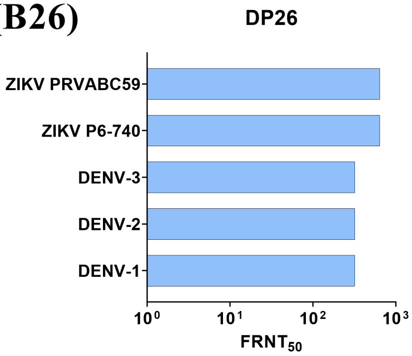

(B27)

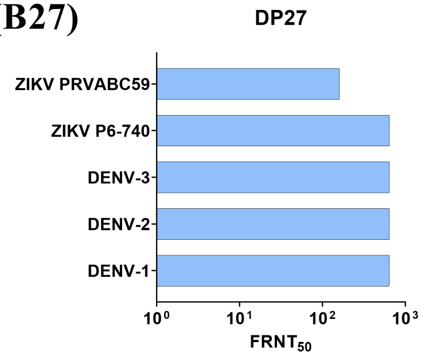

(B28)

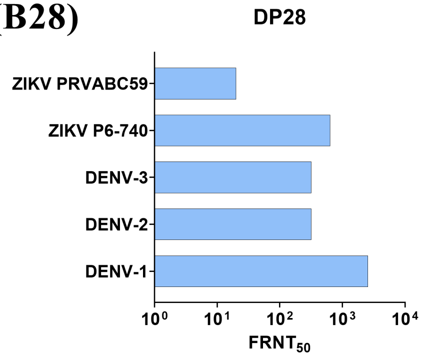

(B29)

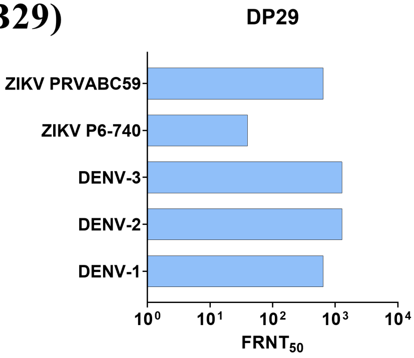

(B30)

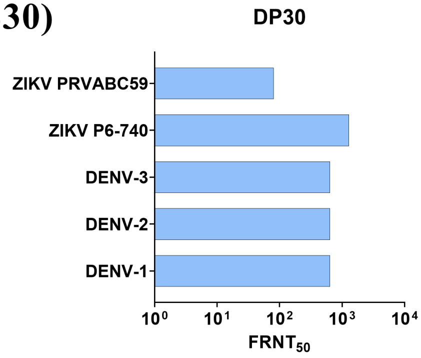

(B31)

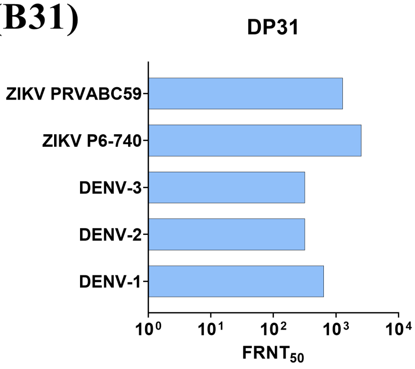

(B32)

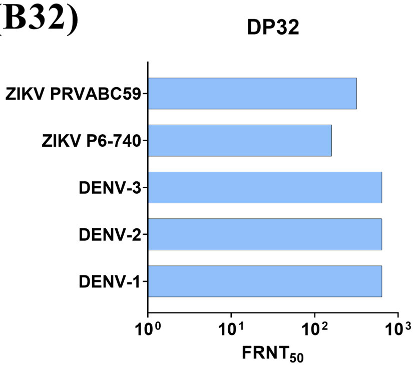

(B33)

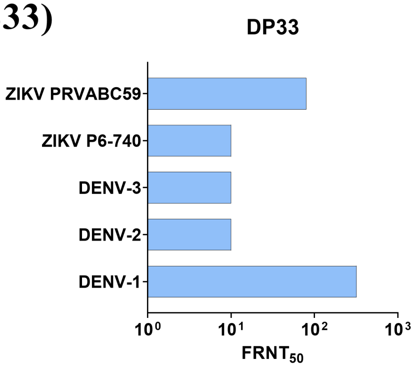

(B34)

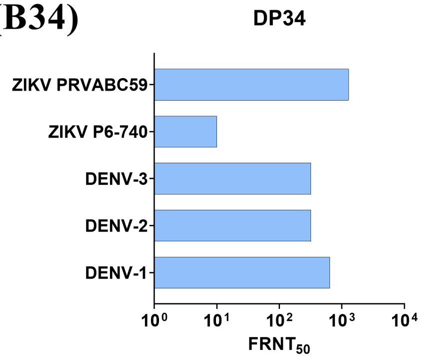

(B35)

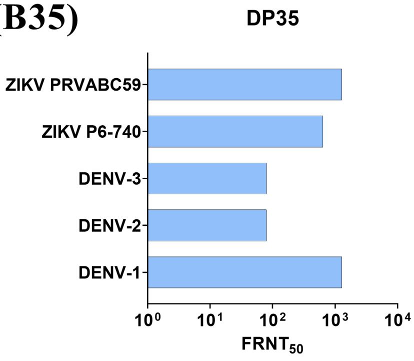

(B36)

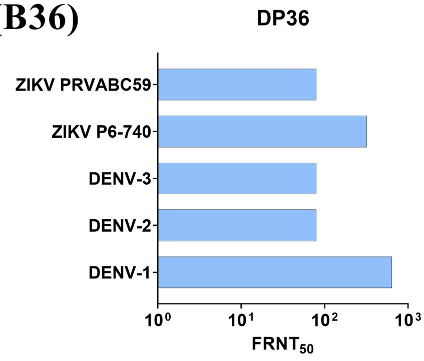

(B37)

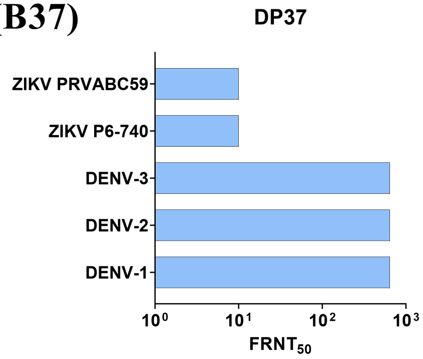

(B38)

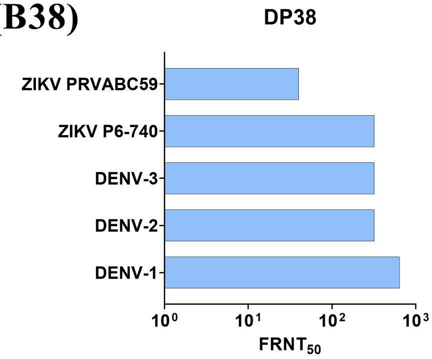

(B39)

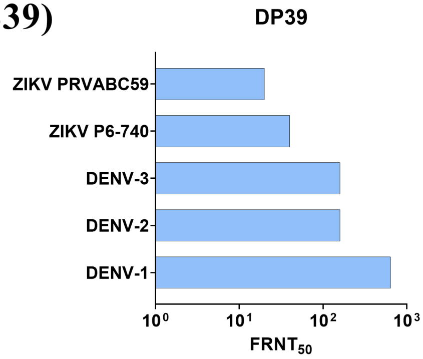

(B40)

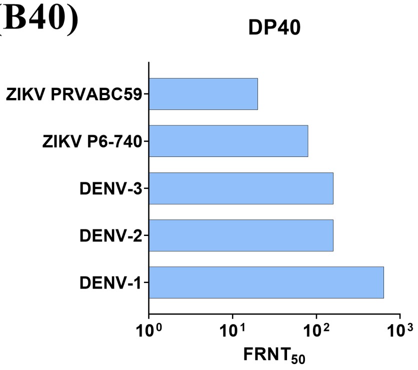

(B41)

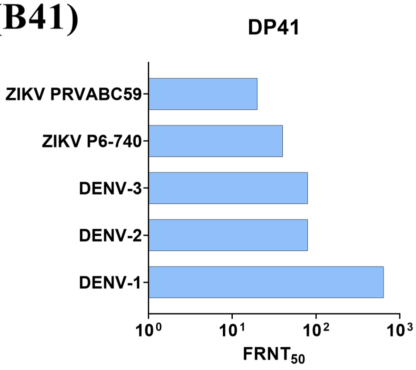

(B42)

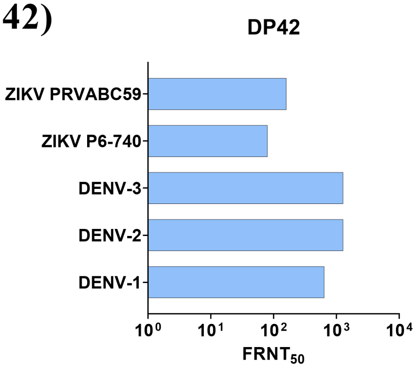

(B43)

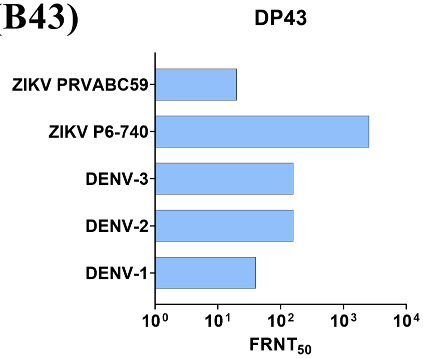

Supplement: Supplementary file 1 [file biomolecules-16-01386-s001.zip › Supplementary Material S2 Individual Serum Neutralization Profiles of the dDENV_IgM-IgG+.pdf]
