# Supplementary material for: Strain-Dependent Cross-Neutralization of Closely Related Asian-Lineage Zika Viruses by Dengue-Immune Sera
Source: Biomolecules. 2026 Sep 23;16(10):1386. doi: 10.3390/biom16101386 (PMC13702172; doi:10.3390/biom16101386)
Supplement: Supplementary file 1 [file biomolecules-16-01386-s001.zip › Supplementary Material S1 Individual Serum Neutralization Profiles of the dDENV_IgM-IgG-.pdf]

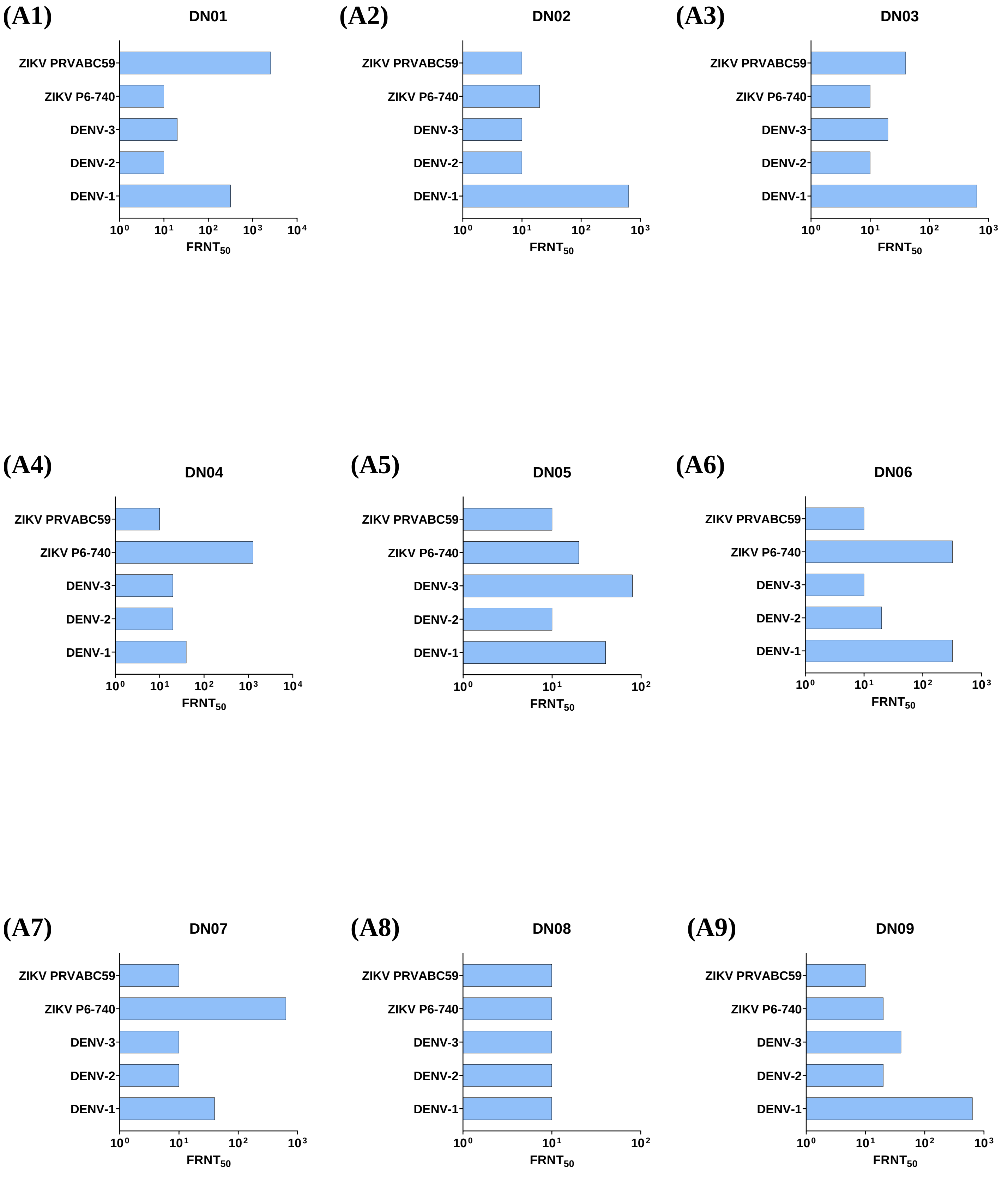

(A1)
DN01
ZIKV PRVABC59
ZIKV P6-740
DENV-3
DENV-2
DENV-1
10^0 10^1 10^2 10^3 10^4
$FRNT_{50}$
(A2)
DN02
ZIKV PRVABC59
ZIKV P6-740
DENV-3
DENV-2
DENV-1
10^0 10^1 10^2 10^3
$FRNT_{50}$
(A3)
DN03
ZIKV PRVABC59
ZIKV P6-740
DENV-3
DENV-2
DENV-1
10^0 10^1 10^2 10^3
$FRNT_{50}$
(A4)
DN04
ZIKV PRVABC59
ZIKV P6-740
DENV-3
DENV-2
DENV-1
10^0 10^1 10^2 10^3 10^4
$FRNT_{50}$
(A5)
DN05
ZIKV PRVABC59
ZIKV P6-740
DENV-3
DENV-2
DENV-1
10^0 10^1 10^2
$FRNT_{50}$
(A6)
DN06
ZIKV PRVABC59
ZIKV P6-740
DENV-3
DENV-2
DENV-1
10^0 10^1 10^2 10^3
$FRNT_{50}$
(A7)
DN07
ZIKV PRVABC59
ZIKV P6-740
DENV-3
DENV-2
DENV-1
10^0 10^1 10^2 10^3
$FRNT_{50}$
(A8)
DN08
ZIKV PRVABC59
ZIKV P6-740
DENV-3
DENV-2
DENV-1
10^0 10^1 10^2
$FRNT_{50}$
(A9)
DN09
ZIKV PRVABC59
ZIKV P6-740
DENV-3
DENV-2
DENV-1
10^0 10^1 10^2 10^3
$FRNT_{50}$

(A10)

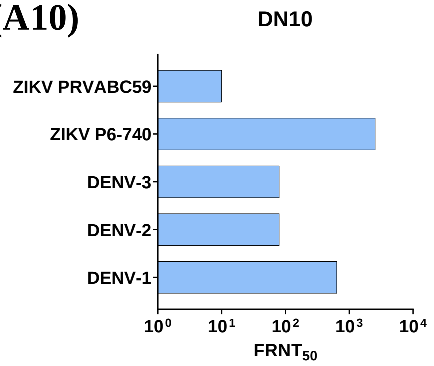

(A11)

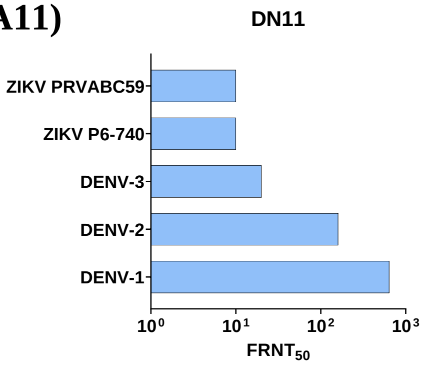

(A12)

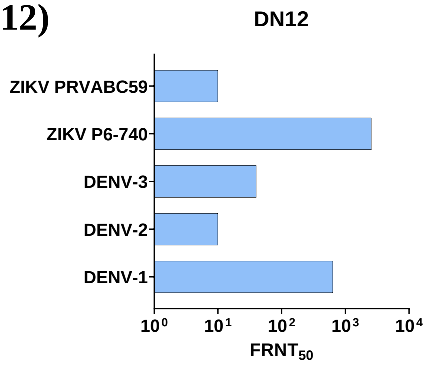

(A13)

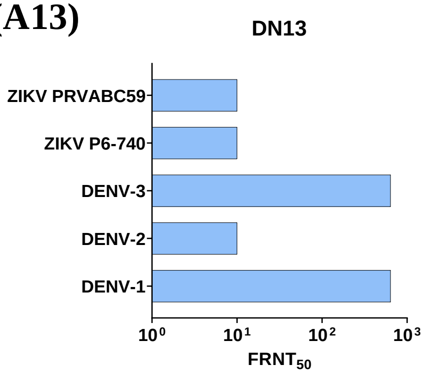

(A14)

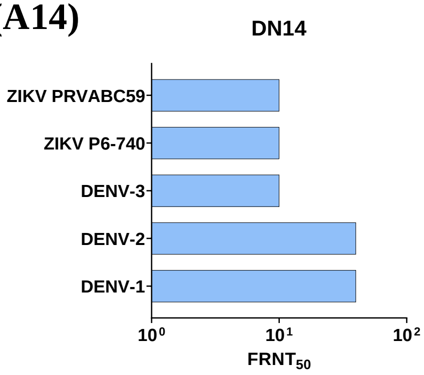

(A15)

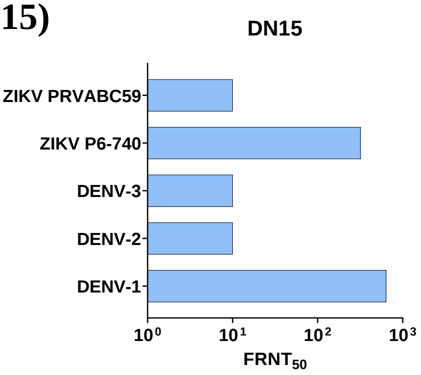

(A16)

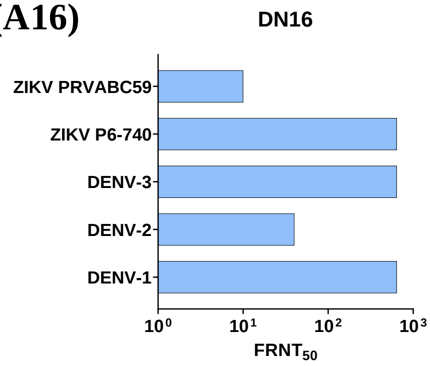

(A17)

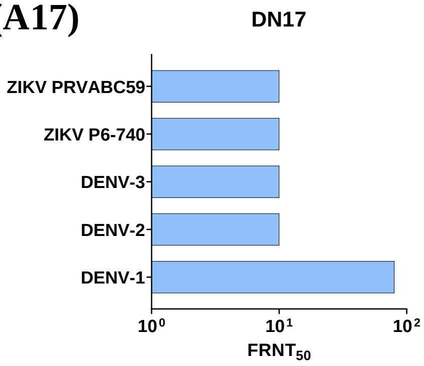

(A18)

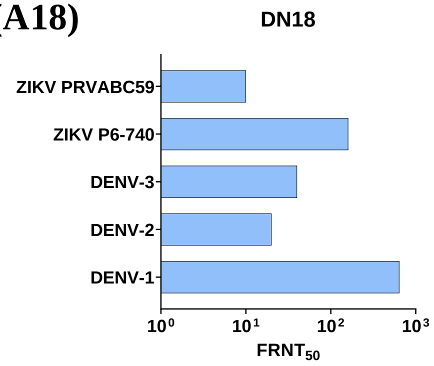

(A19)

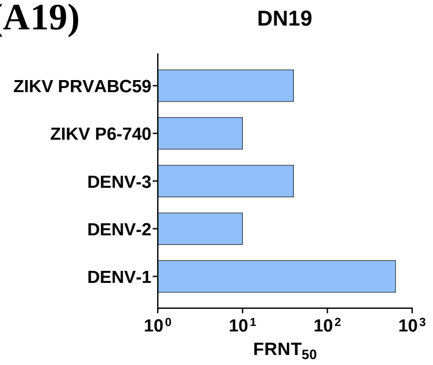
DN19
ZIKV PRVABC59
ZIKV P6-740
DENV-3
DENV-2
DENV-1
10^0
10^1
10^2
10^3
$FRNT_{50}$

(A20)

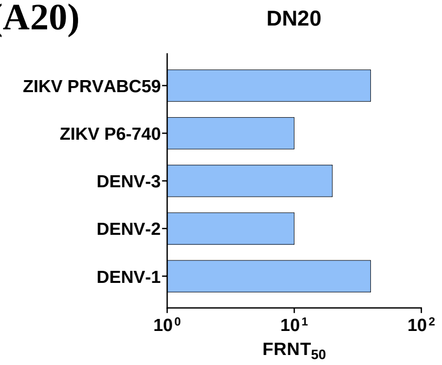
DN20
ZIKV PRVABC59
ZIKV P6-740
DENV-3
DENV-2
DENV-1
10^0
10^1
10^2
$FRNT_{50}$
